# Activation of FGF Signaling Mediates Proliferative and Osteogenic Differences between Neural Crest Derived Frontal and Mesoderm Parietal Derived Bone

**DOI:** 10.1371/journal.pone.0014033

**Published:** 2010-11-18

**Authors:** Shuli Li, Natalina Quarto, Michael T. Longaker

**Affiliations:** 1 Department of Surgery, Children's Surgical Research Program, Stanford University School of Medicine, Stanford, California, United States of America; 2 Department of Structural and Functional Biology, University of Naples Federico II, Complesso M. S. Angelo, Napoli, Italy; Centro Cardiologico Monzino, Italy

## Abstract

**Background:**

As a culmination of efforts over the last years, our knowledge of the embryonic origins of the mammalian frontal and parietal cranial bones is unambiguous. Progenitor cells that subsequently give rise to frontal bone are of neural crest origin, while parietal bone progenitors arise from paraxial mesoderm. Given the unique qualities of neural crest cells and the clear delineation of the embryonic origins of the calvarial bones, we sought to determine whether mouse neural crest derived frontal bone differs in biology from mesoderm derived parietal bone.

**Methods:**

BrdU incorporation, immunoblotting and osteogenic differentiation assays were performed to investigate the proliferative rate and osteogenic potential of embryonic and postnatal osteoblasts derived from mouse frontal and parietal bones. Co-culture experiments and treatment with conditioned medium harvested from both types of osteoblasts were performed to investigate potential interactions between the two different tissue origin osteoblasts. Immunoblotting techniques were used to investigate the endogenous level of FGF-2 and the activation of three major FGF signaling pathways. Knockdown of FGF Receptor 1 (FgfR1) was employed to inactivate the FGF signaling.

**Results:**

Our results demonstrated that striking differences in cell proliferation and osteogenic differentiation between the frontal and parietal bone can be detected already at embryonic stages. The greater proliferation rate, as well as osteogenic capacity of frontal bone derived osteoblasts, were paralleled by an elevated level of FGF-2 protein synthesis. Moreover, an enhanced activation of FGF-signaling pathways was observed in frontal bone derived osteoblasts. Finally, the greater osteogenic potential of frontal derived osteoblasts was dramatically impaired by knocking down FgfR1.

**Conclusions:**

Osteoblasts from mouse neural crest derived frontal bone displayed a greater proliferative and osteogenic potential and endogenous enhanced activation of FGF signaling compared to osteoblasts from mesoderm derived parietal bone. FGF signaling plays a key role in determining biological differences between the two types of osteoblasts.

## Introduction

Bones of the cranial vault form through the process of intramembranous ossification [1]. Calvarial bones arise from two embryonic tissue origins, namely the neural crest and mesoderm. The distinct contributions of each tissue to the skull have been well established by combining mice with a *Wnt1-Cre* construct and a conditional reporter gene, *R26R* [2], [3]. These studies have defined the pattern of cranial neural crest cell migration in mouse embryos and demonstrated that the frontal bone is of neural crest origin, whereas the parietal bone is of mesoderm origin. The neural crest (NC) is a population of cells unique to the vertebrate embryo [4], [5], [6]. NC cell (NCC) progenitors originate from the neural plate border, and migrate into the periphery to contribute to multiple lineages [7], [8], [9]. In all vertebrates a large part of the skull and the entire facial skeleton are derived from cephalic NCC.

Development of the normal skull vault requires mechanisms to ensure that both its morphology and its rate of growth are precisely matched to those of the developing brain. This precise relationship suggests that there are important tissue interactions between the brain and the skeletogenic membranes also involving the mesenchymal layers between them (the developing meninges).

A multitude of signaling molecules, as well as their respective receptors and downstream transcriptional factors, act in concert to regulate bone development [10], [11], [12]. In particular, fibroblast growth factor (FGF) signaling has gained much attention for its major role in skeletogenesis, including calvarial osteogenesis [13], [14], [15], [16]. FGF signaling is known to play a critical role in regulating proliferation and differentiation of osteoblasts and osteogenic precursors [17], [18], [19], [20].

Binding of FGF ligands to their receptors leads to activation of three different intracellular pathways: mitogen-activated protein-kinase (MAPK, including ERKs, p38 and JNKs), Protein Kinase C (PKC) and phosphoinositide 3-kinases (PI3K) [21], [22], [23]. These pathways can mediate effects of FGF-signaling on osteoblast gene regulation [24], [25], [26].

Our previous *in vitro* and *in vivo* studies demonstrated that differences between the neural crest derived frontal and mesodermal derived parietal bone of both, juvenile and adult mice, exist [27]. Of interest, this study revealed that neural crest derived frontal bone has superior potential for osteogenic differentiation and healing compared to mesodermal derived parietal bone. Moreover, an enhanced activation of endogenous canonical Wnt signaling in frontal bone, relatively to parietal bone was identified, both *in vitro* and *in vivo* [27].

Furthermore, a detailed comparative gene expression profile of FGF ligands and their receptors carried out on frontal and parietal bones revealed a differential expression pattern of the major FGF osteogenic genes and their receptors between the neural crestderived frontal bone and the paraxial mesoderm derived parietal bone [28]. Particularly, the expression of ligands such *Fgf-2*, *Fgf-9* and *Fgf-18* was found to be significantly upregulated in frontal bone in embryonic day 17.5 (E17.5), postnatal day 1 and 60 mice. Frontal bone also elaborated higher levels of *Fgf* receptors *FgfR1*, *FgfR2* and *FgfR3* transcripts versus parietal bone. Thus, these observations strongly suggested that neural crest-derived frontal bone might be a highly activated FGF-signaling domain compared to parietal bone. Indeed, determining the role of FGF signaling in imparting superior osteogenic/regenerative potential on calvarial tissue is of paramount importance.

Therefore, our previous studies prompted us to further investigate on the biological properties of neural crest derived frontal and mesodermal derived parietal bones.

In the current study we analyzed the cell proliferation and osteogenic potential properties of embryonic E17.5 E18.5 and early postnatal day 1 (P1) osteoblasts derived from frontal (FOb) and parietal (POb) bones. In addition, we characterized the endogenous activation of the three major FGF signaling pathways: MAPK, PI3K, PKC, and unveiled higher activation of all three FGF signaling pathways in FOb cells compared to POb cells. Furthemore, by knocking down the *Fgf Receptor1*(*FgfR1*) we have highlighted the crucial role played by FGF signaling in conferring greater osteogenic potential to neural crest derived frontal osteoblasts.

## Materials and Methods

### Animals

All experiments using animals were performed in accordance with Stanford University Animal Care and Use Committee guidelines with protocol ID #8397. CD-1 wild-type mice were purchased from Charles River Laboratories Inc., Wilmington, MA. Animals were housed in light- and temperature-controlled rooms and were given food and water *ad libitum*.

### Tissue Harvesting and Primary Cell Culture

Neural crest derived frontal osteoblasts (FOb) and mesoderm derived parietal osteoblasts (POb) were harvested from skulls of embryonic day 17.5 (E17.5), 18.5 (E18.5) and postnatal day 1 (P1) respectively. The periosteum and dura mater were carefully stripped off from the skull. The peripheral suture complexes of each frontal or parietal bone were also carefully removed. Frontal and parietal bones were minced separately into small chips less than 1 mm before digestion. Embryonic skulls (E17.5 and E18.5) were digested with 0.1% Collagenase A (Roche Diagnostics, Indianapolis, IN, USA) in serum free α-MEM (alpha minimum essential medium, Gibco Life Technologies and Invitrogen Corporation, Carlsbad, CA) for 30 minutes at 37°C in a shaking water bath. P1 skulls were digested with 0.2% Dispase II and 0.1% Collagenase A (Roche Diagnostics, Indianapolis, IN, USA) in serum-free medium. The digestion was carried out 6 times, each 10 minutes. The first 2 digestions were discarded. The later four digestions were pooled together. All digestions were neutralized with an equal volume of α-MEM supplemented with 10% FCS (fetal calf serum, Gemini Bioproducts, Woodland, CA), 100 IU/ml penicillin and streptomycin (Invitrogen Corporation), pelleted, and resuspended in the culture medium. Both FOb and POb cells were plated in 100 mm tissue culture dishes (Corning Incorporated, San Mateo, CA) and incubated at 37°C with continuous supplement of 5% CO_2._ The medium was changed every other day. Only passage 0 and 1 cells were used for all experiments.

### FOb and POb cell co-cultures

Co-culture experiments were carried out using 6-transwell chamber plates (Corning Costar, Cambridge, MA). FOb or POb were plated onto either on the top chambers (inserts) (4×10^4^/insert for 6 well plates & 2×10^4^/insert for 12 well plates) or the bottom chambers (8×10^4^/well for 6 well plates & 4×10^4^/well for 12 well plates). The inserts had a pore size of 0.4 µm. This pore size would allow the diffusion of soluble factors between the two cell populations, but would prevent the transfer of any cells or organelles. The two cell populations were cultured separately overnight and then were combined with regular culture medium: α-MEM supplemented with 10% FCS, 100 IU/ml penicillin and 100 IU/ml streptomycin. For cell proliferation assay, the combined two cell populations of the 12 well plates were cultured with serum free α-MEM for 12 or 24 hours and the cells were labeled with BrdU for 12 hours and detected. For cell differentiation assay, cells were incubated with the osteogenic differentiation medium (ODM), α-MEM supplemented with 10 µM glycerol β-phosphate, 0.25 µM ascorbic acid, (Sigma-Aldrich, MO, USA), 1% penicillin, streptomycin and 3% FCS. The bottom chambers were stained for alkaline phosphatase at day 7 of differentiation assay using the Alkaline phosphatase Kit (Sigma-Aldrich, MO, USA) according to the manufacturer's instructions. Alkaline phosphatase enzymatic activity was determined by biochemical colorimetric assay with Sigma-Aldrich alkaline phosphatase staining kit (85L-2) as previously described [20]. After three weeks of differentiation cells were stained with Alizarin red staining, followed by its quantification as previously described [27], [29].

### Statistical Analysis

The results are presented as the mean ± SD of three independent experiments. Statistical differences between the means are examined by Student's test. The comparison for significant differences in multiple groups was done with ANOVA. A *P* value <0.05 was considered statistically significant.

### Osteogenic Differentiation Assays

FOb or POb cells were plated in 6-well-plate (1×10^5^/well). Upon sub-confluence, the osteogenic differentiation medium (ODM, 10 µM glycerol β-phosphate, Sigma and 0.25 µM ascorbic acid, Sigma in α-MEM supplemented with 1% penicillin, streptomycin and 10% FCS) was applied onto cells. The medium was changed every other day. The ODM made by frontal osteoblast cell-conditioned medium (dFCM) or by parietal osteoblast cell-conditioned medium (dPCM) were supplemented with 3% FCS. Alkaline phosphatase and Alizarin red staining to assess mineralization of extracellular matrix were performed as above. All microscopic observations and images capturing were conducted by using Leica DMIL microscope and Leica Microsystems digital imaging software (Leica Microsystems Wetzlar, Germany).

Procedures for RNA isolation, reverse-transcriptase polymerase chain reaction (RT-PCR), quantitative real time PCR analysis and primer sequences were previously described [20], [27], [29].

### Preparation of Cell-Conditioned Media

Cell-conditioned media (CM) were obtained from either FOb or POb cells. The two conditioned medium are referred as frontal conditioned medium (FCM) and parietal conditioned medium (PCM). Briefly, 8×10^5^ FOb or POb cells (8×10^5^) were plated in 100 mm plates separately. Upon subconfluence, the cells were washed three times with sterile PBS prior to addition of serum free medium. After culture in serum free medium for 24 hours, the cell-conditioned media were collected and concentrated 10 fold using Centricon filters (Centricon-3, 3000 NMWL, Millipore Corporation, MA). Collection and concentration of the media were carried out at 4°C. The concentrated media were replenished with fresh α-MEM to the original volumes. The volumes of the conditioned media were also normalized by cell numbers so that an equal volume of FCM or PCM was produced by equal number of FOb or POb cells. All experiments were performed three times using freshly harvested media.

### Cell Proliferation Assays

The growth rate of FOb and POb cells was assessed by BrdU incorporation assay as well as immunoblotting analysis of proliferating cellular nuclear antigen (PCNA). For the BrdU incorporation assay, FOb and POb cells were plated at density of 1500 cells/well, in 96-multiwell culture plates with flat bottoms (Corning Incorporated, San Mateo, CA). Cells were incubated in culture medium for 6 hours. Then, cells were washed twice with sterile PBS and cultured in serum-free α-MEM or FCM and PCM for 12 or 24 hours. BrdU incorporation was carried out for 12 hours (Roche Diagnostics Corporation, IN) according to the manufacturer's instructions. Photometric detection was done with an ELISA reader at 370 nm wavelength. The background was subtracted when the resulting data were processed. Each experiment was run in triplicate. For the growth curve assay, FOb and POb cells were seeded in 6 well plates in triplicate at density of 10^5^ cells/well. Cells were grown in α-MEM supplemented with 1% penicillin, streptomycin and 10% FCS. Cell counting was performed in triplicate using a hematocytometer at days 2, 4, 6, 8 and 10. The first day after seeding represents day 0. For the growth curve with inhibitors cells were plated and cultured as above. Growth medium was supplemented with 5 µM U-0126, 10 µM LY-294002 and 1 µM GÖ-6983. Control cells were maintained in growth medium containing 0.1%DMSO. Western blotting analysis of PCNA was performed on cell lysates, using RIPA Buffer (Sigma Aldrich, US). Total cellular proteins were extracted from FOb and POb after cultured in serum-free medium for 24 hours. Proteins were quantified by Bicinchoninic Acid (BCA) protein assay (Pierce, IL). Fifty micrograms of total cellular protein were resolved onto 10% Nupage Tris-HCl sodium dodecyl sulfate (SDS)-PAGE gel (Precast Nupage gel, Invitrogen, Life Technologies) and transferred onto Immuno-Blot PVDF membranes (Bio-Rad, Hercules CA, USA). Membranes were blocked with 5% natural nonfat dry milk before probed with anti-PCNA antibody (FL-261 sc7970, rabbit polyclonal 1∶400, Santa Cruz Biotechnology, CA, USA). A horseradish peroxidase-conjugated anti-mouse (HRP) 1∶8000, (Santa Cruz Biotechnology, CA) was used as secondary antibody. Immunoblotted products were visualized by enhanced chemiluminescence substrate (AmershamBiosciences, UK). Subsequently, the membranes were stripped of the antibodies by incubation onto a stripping solution (62.5 mM Tris-HCl, pH 7.5, 2%SDS, 100 mM β-mercaptoethanol) for 30 minutes at 52°C followed by three washes at RT with 20 mM Tris, 136 mM NaCl pH 7.6 (TBS). Membranes were blocked with 5% milk and then incubated with α-tubulin antibody (B-7 sc-5286, mouse monoclonal 1∶600, Santa Cruz Biotechnology, CA) to control for equal loading and transfer of the samples.

### Detection of FGF-2 protein in FOb and POb cells

Endogenous FGF-2 was detected by western blot analysis. Cell lysates were prepared with RIPA buffer and the total proteins were quantified by BCA assay as mentioned above. Fifty micrograms of total cellular protein were separated onto 12% Nupage gels Tris-HCl sodium dodecyl sulfate (SDS)-PAGE, transferred onto Immuno-Blot PVDF membranes, and blocked with 5% natural nonfat dry milk as described before. FGF-2 was detected by anti-FGF-2 antibody (147, SC-79, rabbit polyclonal 1∶400, Santa Cruz Biotechnology, CA, USA) and a horseradish peroxidase-conjugated anti-rabbit antibody (1∶8000, Santa Cruz, Biotechnology, CA, USA) was used as secondary antibody Densitometry of FGF-2 bands was performed using the ImageJ software program, (NIH, Bethesda, MA). The density of each FGF-2 bands was normalized to the loading controls (α-tubulin).

### Neutralization of FGF-2 Bioactivity

A polyclonal goat anti-FGF-2 antibody (R&D systems, MN) was used for FGF-2 neutralization. Serum-free FCM and PCM were pre-incubated with 1 µg/ml, 2 µg/ml or 4 µg/ml of anti-FGF-2 antibody for 3 hours at 4°C prior to application on FOb or POb cells. Osteoblast proliferation was determined by BrdU incorporation assay as described above. Osteoblasts stimulated with 20 ng/ml of rhFGF-2 (hBA-154, Santa Cruz, Biotechnology, CA, USA) were used as positive controls. Normal irrelevant rabbit IgG (sc2027, Santa Cruz Biotechnology, CA, USA) was used as internal control, and serum-free medium as negative control.

### Characterization of activation of FGF Signaling Pathways in FOb and POb cells

The endogenous activation of FGF signaling pathways in FOb and POb cells was investigated by immunoblotting analysis of phosphorylated signaling proteins. Subconfluent FOb and POb cells were washed twice with 1X PBS and starved in serum-free medium overnight. Then the cells were washed twice with ice-cold PBS and lysed with cold lysis buffer (50 mmol/L of HEPES, pH 7.5, 150 mmol/L of NaCl, 1 mmol/of EDTA, 10% glycerol, 1% Triton-X-100, 25 mmol/L of sodium fluoride) containing 1 mmol/L of sodium orthovanadate and Protease Inhibitor Cocktail (Sigma-Aldrich, St. Louis, MO). Cell lysates were assayed for protein concentration by BCA assay as described above. Aliquots (50–100 µg) of cell lysate were electrophoresed on 12% Tris-HCl sodium dodecyl sulfate (SDS)-PAGE gels (Precast Nupage gels, Invitrogen, Life technologies) and transferred onto Immobilon-P membrane (Millipore Corporation, Bedford, MA). Antibodies against the specific phosphorylated proteins of each signaling pathway were chosen as follows: anti-phospho-ERK p44/42 (Thr202/Tyr204 rabbit polyclonal antibody 1∶1000, Cell Signaling Technology, Beverly, MA), anti-phospho-Akt (Ser 473 rabbit monoclonal antibody 1∶1000, Cell Signaling Technology, Beverly, MA), anti-phospho-PKC α/β Thr638/641 rabbit polyclonal antibody 1∶1000, Cell Signaling Technology, Beverly, MA), anti-phospho-PKC δ (Thr505 rabbit polyclonal 1∶1000, Cell Signaling Technology, Beverly, MA), anti-ERK-1/2 (C-14 mouse monoclonal antibody 1∶400, Santa Cruz Biotechnology, Inc., Santa Cruz, CA), anti-Akt (rabbit polyclonal antibody 1∶1000, Cell Signaling Technology, Beverly, MA) and anti-PKC/PKD (rabbit polyclonal antibody 1∶1000, Cell Signaling Technology, Beverly, MA). A horseradish peroxidase-conjugated anti-rabbit antibody (HRP) 1∶8000 was used as secondary antibody. Immunoblotted products were visualized by enhanced chemiluminescence substrate (Amersham Biosciences, UK). α-tubulin antibody was used to control for equal loading and transfer of the samples, as mentioned previously. All bands of the phosphorylated or non-phosphorylated proteins in the immunoblots were normalized with the loading controls (α-tubulin) and quantified by densitometry. The statistical significance in comparison of the 2 type cells were presented by asterisks when P values were less than 0.05.) The FGF-2 induction and inhibitor blocking of the three signaling transduction pathways were also conducted on embryonic E17.5 cells (FOb & POb). 20 ng/ml of exogenous FGF-2 was used for the induction (rhFGF-2, SantaCruz Biotechnology). Inhibitors to block the three FGF signaling pathways) block were used as follows: 10 µM U-0126 to block the MAPK pathway, 20 µM LY-294002 to block the PI3K pathway, and 2 µM GÖ-6983 to block the PKC pathway (Calbiochem-Novabiochem. The inhibitors were dissolved in 0.1% DMSO as vehicle. Briefly, subconfluent cells were starved at in serum-free medium overnight and pre-incubated for 30 minutes with one of the three inhibitors before any treatments. The rhFGF-2 induction was carried out for 30 minutes. To determine the nonspecific contribution of DMSO to FGF-2 signal inhibition, a separate group of cells was incubated with 0.1% DMSO alone. A group of cells without FGF-2 induction was also incubated with the inhibitors for 30 minutes to block the phosphorylation of the endogenous signaling proteins. Cell lysate preparations, immunoblotting analysis and densitometry were performed as described above. All experiments were performed 3 times with similar results. The densitometric results of both phosphorylated and non-phosphorylated signaling proteins were normalized to the respective loading control (alpha-tubulin bands).

### Transduction of FOb cells with shFgfR lentivirus

Primary E17.5 osteoblasts (passage 0) derived from frontal bone were transduced with *FgfR1* shRNA lentiviral particles (Flg shRNA sc-29317-V, Santa Cruz Biotechnology) as previously described [27], [29]. A lentivirus shRNA Scramble (sc-108080, Santa Cruz Biotechnology) was used as negative control. After 24 hours, the infected cells were split 1∶4 and grown in α-MEM supplemented with 10%FCS and 10 µg/ml Puromicin. Upon puromycin selection, puromycin positive cells were seeded in 6-well plates at concentration of 380.000/well and cultured in osteogenic medium (α-MEM supplemented with 10%FCS, 10 mM β-glycerophosphate and 100 µg/ml ascorbic acid). To validate the downregulation of *FgfR1*, RNA was isolated from cells and reverse transcribed as previously described [20]. Conditions for PCR analysis and oligos sequence were previously described [27]. Immunoblotting analysis was performed on 100 µg of cell lysate using anti-FGFR1 antibody (Flg C-15/sc-121, dilution 1∶200) Alkaline phoshatase enzymatic activity and Alizarin staining were performed as mentioned above. Experiments were performed two times with similar results.

## Results

### Osteogenic potential of neural crest derived frontal osteoblasts and mesenchymal derived parietal osteoblasts

**Figure 1:**
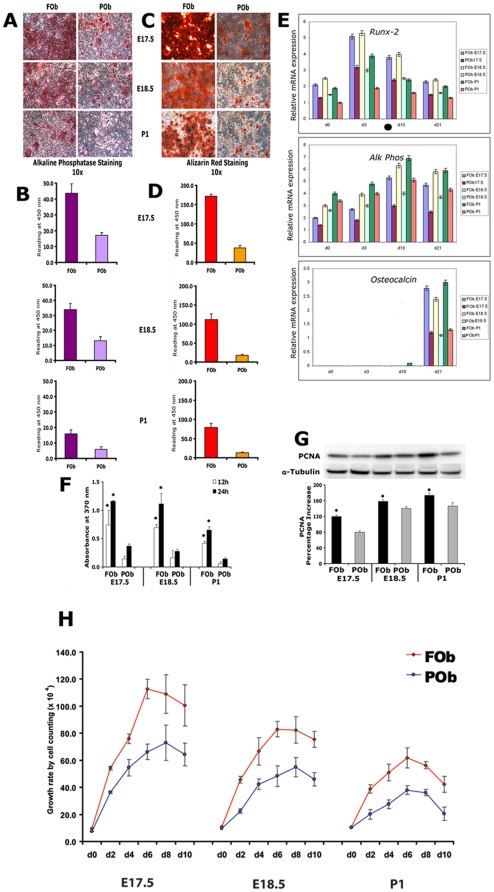
Osteogenic and mitogenic potential of FOb and POb cells. **A**, alkaline phosphatase staining performed at day 7 of osteogenic differentiation on frontal osteoblasts (FOb) and parietal osteoblasts (POb) harvested from embryonic 17.5 and 18.5 day (E17.5, E18.5) and post-natal day (P1) mice. **B**, Alkaline phosphatase enzymatic activity. **C**, mineralization of extracellular matrix was assessed by Alizarin Red staining at day 21 day. **D**, quantification of Alizarin red staining. Both alkaline phosphatase activity and mineralization of extra cellular matrix were more robust in FOb than in POb, thus indicating the higher osteogenic capacity of the neural crest derived osteoblasts. **E**, quantitative real time PCR of osteogenic markers *Runx-2*, *Alk-Phos* and *Osteocalcin* **F**, cell growth of E17.5, E18.5, and P1 FOb and POb cells was assessed by BrdU incorporation assay at 12 or 24 hours in serum free medium cultures. **G**, cell growth investigated by immunoblotting analysis of proliferating cellular nuclear antigen (PCNA). Cell lysates were collected after 24 hours culture in serum-free (SF) medium. α-tubulin was used as loading control. Histogram (bottom panel) represents the densitometric analysis of the electrophoresis bands. **H**, long term growth curve assay performed by cell counting. The values are presented as means ± SD of three independent experiments. BrdU incorporation, PCNA and growth curve assays showed statistical significant higher cell proliferation capability in FOb as compared to POb. The results are presented as the mean ± SD of three independent experiments. Asterisk * represent p<0.05.

To verify whether differences in osteogenic potential of neural crest derived and mesodermal derived calvarial bones could be already detect at embryonic stages we performed an osteogenic differentiation assay on FOb and POb cells harvested from embryos at day 17.5 and 18.5. P1 FOb and POb cells were used as reference. Staining for Alkaline phosphates, an early/intermediate marker of osteogenesis, revealed striking differences between FOb and POb cells. As illustrated in **Figure 1A and B**, at day 7 of differentiation E17.5 and E18.5 FOb cells showed a more intense alkaline phosphatase staining and enzymatic activity than POb cells, indeed, resembling differences previously observed between either juvenile or adult FOb and POb [27]. Alizarin Red staining and its quantification (Figure 1C and D) revealed even more striking differences between embryonic FOb and POb cells, larger bone nodule formation and more robust mineralization of extracellular matrix could be detected in FOb as compared to POb (**Figure 1C**). To further examine the osteogenic potential of frontal and parietal osteoblasts, the temporal expression of early, intermediate, and late osteoblast specific markers *Runx-2*, *Alk phos*, and *Osteocalcin* was determined by quantitative real time RT-PCR analysis (Figure 1E). This analysis revealed higher expression levels of all three osteogenic markers in FOb cells as compared to POb cells. Thus, the above data indicated that indeed, differences in the osteogenic potential of FOb and POb cells could be already identified in embryonic tissues.

To further delineate differences between the two types of osteoblasts we next analyzed their cell proliferation by BrdU incorporation assay. As shown in **Figure 1E**, FOb cells displayed significant higher cell proliferation capabilities compare to POb cells. The higher proliferation activity of FOb was further confirmed by western blot analysis of proliferating cellular nuclear antigen (PCNA) revealing a more intense PCNA bands in FOb (**Figure 1F**). The results were normalized to α-tubulin. Each electrophoresis band was quantified by densitometry, the differences between the FOb and POb cells were statistically significant with a *P value less than 0.05.

In addition, a long term growth curve assay performed by cell counting for 10 days also revealed a greater proliferative activity in FOb cells as compared to POb cells (Figure 1H).

### Interactive effects of neural crest derived frontal osteoblasts and mesenchymal derived parietal osteoblasts on cell growth and osteoblast differentiation

**Figure 2:**
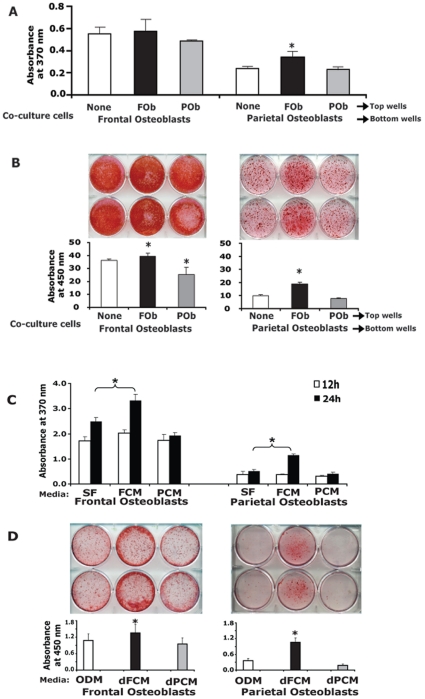
Effects of interactions between FOb and POb cells on cell proliferation and osteoblast differentiation. **A**, BrdU incorporation assay indicated that cell proliferation was significantly induced in POb cells when co-cultured with FOb cells. **B**, osteoblast differentiation of FOb and POb co-cultures was investigated by Alizarin red staining and its quantification. Osteogenic differentiation of POb cells was significantly enhanced when co-cultured with FOb cells, while co-cultures of FOb or POb cells with POb cells showed negative effects. Cells cultured alone were used as controls. **C**, BrdU incorporation of FOb and POb cells cultured with FOb- and POb- derived conditioned media (FCM & PCM, serum free) revealed that FMC significantly increased cell proliferation on both, FOb and POb cells at 24 hours. Serum free medium was used as control. **D**, osteogenic differentiation of FOb and POb cells in presence of differentiation –FCM (dFMC) and differentiation-PCM (dPMC) supplemented with 3% FCS, was measured and quantified by Alizarin Red staining. A greater osteogenic differentiation was observed in both FOb and POb cells only when incubated with dFCM, but not with dPCM. Osteogenic differentiation medium supplemented with 3% FCS was used as control.

To investigate whether FOb and POb influence each other in their proliferation and osteogenic potential we performed co-cultures using the two types of cells harvested from E17.5 mice. In co-cultures, FOb and POb were separated by a micro-porous membrane with 0.4 µm pores, so that only humeral factors released in the medium but not cells, could pass through the membrane. Two different types of co-culture were set up: heterotypic culture comprising of FOb and POb and homotypic cultures comprising of FOb/FOb and POb/POb. Each cell type cultured alone was used as control. Measurement of cell proliferation by BrdU incorporation clearly indicated that POb cells proliferated more than control when co-cultured with FOb, but not when co-cultured with POb cells (**Figure 2A**). Moreover, other combinations of co-cultures did not show significant mitogenic effect. The osteogenic differentiation of FOb and POb in co-culture was also tested (**Figure 2B**). After 3 weeks of differentiation, Alizarin Red staining assay and its quantification was performed on the osteoblasts in the lower compartment of the transwell plate. Overall, POb cells differentiated less than FOb cells. Interestingly, for POb cells, the co-culture with FOb significantly promoted the osteogenic differentiation. Homotypic FOb co-cultures did not show prominent effect on differentiation, while the co-cultures with POb appeared to have a slight inhibitory effect on FOb differentiation.

Thus, results from the co-culture experiments indicated that there are humeral factors released in the medium by FOb which might in a paracrine fashion promote proliferation and/or osteogenic differentiation of POb. To explore this possibility we collected conditioned media, from both frontal (FCM) and parietal osteoblasts (PCM), and tested it on the two cell types. As assessed by BrdU incorporation, a significant mitogenic effect was exerted by frontal cell-conditioned medium (FCM) on both frontal and parietal osteoblasts (**Figure 2C**). Conversely, the parietal cell-conditioned medium (PCM) did not elicit any mitogenic either on FOb or POb cells. Moreover, the FCM enhanced osteogenic differentiation of FOb and POb, and the effect was more dramatic on POb as revealed by Alizarin red staining and its quantification (**Figure 2D**).

### Differential levels of biological FGF-2 protein between primary cultures of neural crest derived frontal osteoblasts and mesenchymal derived parietal osteoblasts

**Figure 3:**
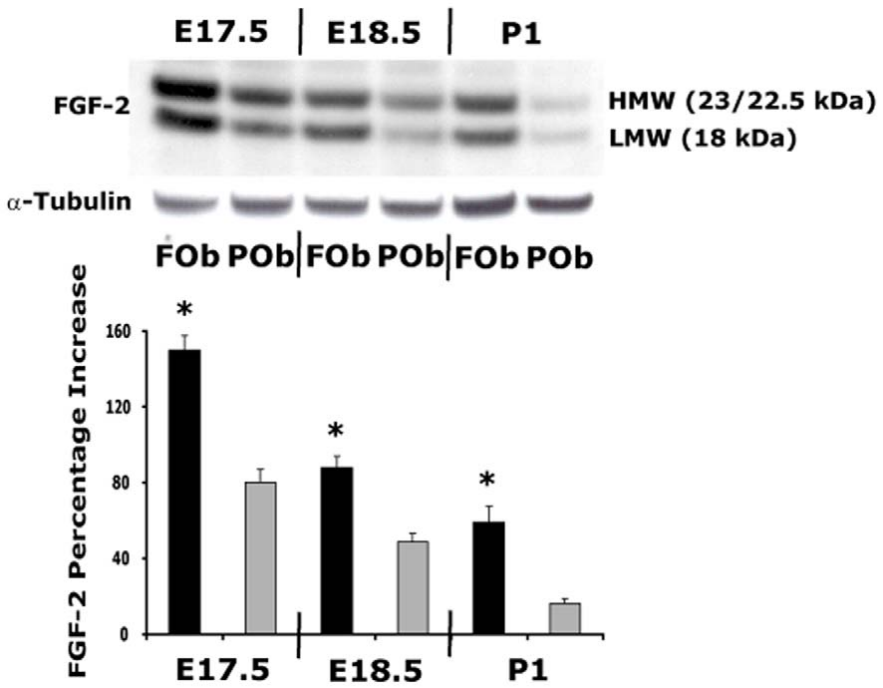
Identification of cellular FGF-2 proteins in neural crest derived FOb and mesenchymal derived POb cells. Endogenous levels of FGF-2 were analyzed on FOb and POb cells by immunoblotting using antiFGF-2 antibody. Higher levels of high molecular weight (HMW) and low molecular weight (LMW) FGF-2 forms were observed in FOb compared to POb at all stages analyzed. The total cellular FGF-2 proteins were quantified by densitometry (lower panel), α-tubulin was used as loading control. Histogram represents the densitometric analysis of electrophoresis bands, the relative intensities of bands were normalized to their respective loading control and set as 100% The results are presented as the mean ± SD of three independent experiments. The asterisks represent P value <0.001.

FGF-2 is one of the well-known mitogens and widely reported to be involved in osteogenesis. Indeed, it could also be one of the factors contributing to the differences in proliferation and osteogenesis observed between FOb and POb cells. Therefore, to test this hypothesis we performed a western blotting analysis on FOb and POb. Immunoblotting using anti-FGF-2 antibody identified both high (HMW) and low molecular (LMW) weight FGF-2 forms, and demonstrated clear differences between FOb and POb cells (**Figure 3**). Significantly higher levels of FGF-2 proteins were detected in FOb cells compare to POb cells. This trend was observed from embryonic stages through postnatal day1, with highest difference in FGF-2 protein levels occurring in E17.5 FOb cells.

**Figure 4:**
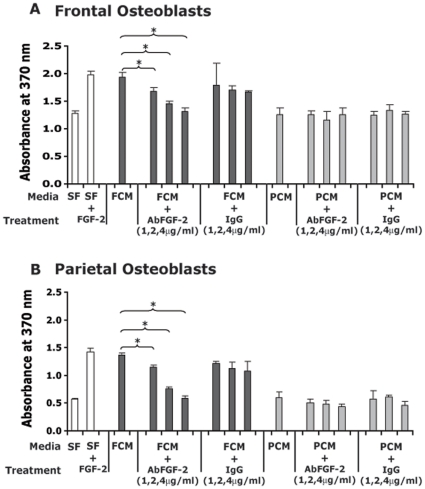
Biological effect of exogenous FGF-2 on cell proliferation of FOb and POb cells. Cell proliferation of FOb and POb harvested from E17.5 mice was measured by BrdU. Osteoblast cells were cultured with conditioned serum-free media collected from frontal osteoblasts (FCM) or parietal osteoblasts (PCM) after 24 hours culture. The conditioned media were incubated with neutralizing anti-FGF-2 antibody at different concentrations (1, 2 and 4 µg/ml). Same concentrations of irrelevant rabbit IgG were used as control. 20 ng/ml of hrFGF-2 was added to the medium as a positive control (SF + FGF-2) and serum-free medium as a negative control (SF). FCM induced significant mitogenic effect, as much as the exogenous added rhFGF-2, on both FOb and POB cells. Anti-FGF-2 antibody blocked the FGF-2 mitogenic effect in a dose-dependent manner. Conversely PCM did not elicit mitogenic effect either on FOb or POb cells and there was no blocking effect by the FGF-2 neutralizing antibody on PCM.

The above results indicated that neural crest-derived FOb cells expressed higher levels of both HMW and LMW FGF-2 forms. This observation would strongly suggest that the FGF-2 could be responsible for the strong mitogenic effect elicited by FCM (see **Figure 2C**). To verify this hypothesis, we performed a cell proliferation assay using FCM incubated with neutralizing anti-FGF-2 antibody or FCM alone. For this purpose, FOb and POb cells were cultured either in FCM or PCM for 24 hours and the cell growth was measured by BrdU incorporation. Recombinant human FGF-2 protein (rhFGF-2) at concentration of 20 ng/ml was used as a positive control and normal irrelevant rabbit IgG as a negative control. As illustrated in **Figure 4**, FCM alone induced strong mitogenic activity on both FOb and POb cells, almost similar to that elicited by rhFGF-2 (SF + FGF-2) stimulation, as compared to serum-free medium control (SF). Incubation with anti-FGF-2 antibody (FCM + Ab-FGF-2) inhibited significantly the mitogenic activity of FMC. The inhibitory effect of neutralizing anti-FGF-2 antibody was dose-dependent. In contrast, PCM did not induce any mitogenic effect either on FOb or POb cells, and no blocking effect by neutralizing antibody on PCM (PCM + FGF-2 Neutralizing Ab) was observed. The latter observation would suggest that there was no substantial amount of FGF-2 present in PCM. Moreover, the irrelevant normal IgG (FCM or PCM + IgG) did not show significant inhibitory activity on the mitogenic effect of either FCM or PCM. Taken together, these experiments suggested that the major mitogenic activity of FOb cells and FCM was at least in part driven by FGF-2.

### Differential activation of FGF signaling pathways between neural crest derived frontal osteoblasts and mesenchymal derived parietal osteoblasts

**Figure 5:**
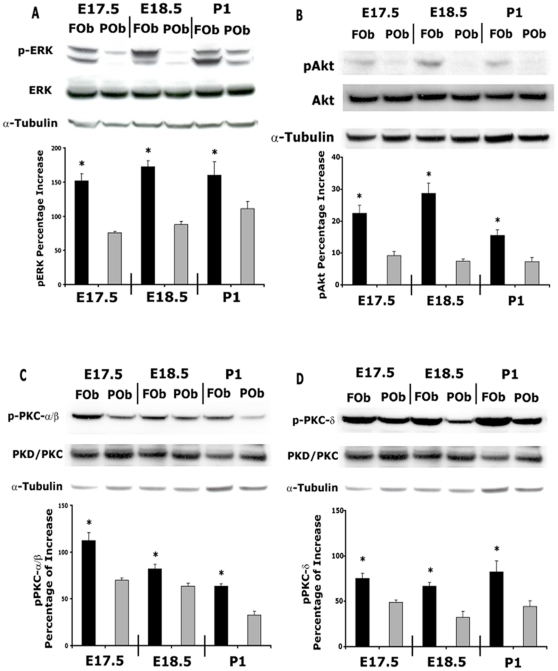
Differential activation of FGF signaling pathways between FOb and POb cells. **A**, endogenous phosphorylated ERK protein of MAPK pathway was assessed by immmunoblotting using specific pERK antibody. Total ERK protein levels were analyzed using a pan-ERK antibody. Significantly higher levels of pERK protein were detected in FOb cells compared to POb cells. **B**, phosphorylated Akt of PI3K pathway was determined with pAkt antibody. Total Akt protein was analyzed with pan-Akt antibody. **C**, phosphorylated PKC α/β and **D**, PKC δ of PKC pathway were investigated by immunoblotting analyses using specific antibody against the phosphorylated proteins Total levels of PKC α/β and PKC δ proteins were detected using antibody against non-phosphorylated proteins. FOb cells displayed significantly higher endogenous activation of all three FGF signaling pathways as compare to POb cells. Each membrane was stripped and subsequantially incubated with non-phosphorylated ERK, Akt, PKC α/β, PKC δ and α-tubulin antibody to assess for the total amount of endogenous proteinsand to control for equal loading and transfer of the samples. Histograms represent the densitometric analysis of electrophoresis bands, the relative intensities of bands were normalized to their respective loading control and set as 100% The results are presented as the mean ± SD of three independent experiments. Asterisk * represents statistical significance (*p<0.05).

Thus, the above data revealed differential levels of FGF-2 protein between FOb and POb cells. This observation would suggest that a differential endogenous activation of FGF signaling pathways might also exist. Three FGF signaling transduction pathways have been widely reported in the literatures: MAP kinase, PI3K and PKC pathways [24], [25], [30]. To verify this hypothesis we investigated the endogenous levels of phosphorylated FGF signaling proteins on FOb and POb cells by immunoblotting analysis at early stage of osteogenesis (**Figure 5A–C**). As illustrated in **Figure 5A**, FOb cells of E17.5, E18.5 and P1 mice had significant higher levels of phosphorylated ERK protein (p-ERK).

Similarly, for the PI3K signaling pathway, sharp differences were observed between FOb and POb cells, with FOb cells displaying significantly higher endogenous levels of phosphorylated Akt protein (p-Akt) (**Figure 5B**).

Finally, the endogenous levels of phosphorylated PKC α/β and PKC δ signaling proteins of PKC pathway was analyzed (**Figures 5C and D**). Significantly higher levels of phosphorylated PKC α/β & PKC δ proteins, were detected in FOb compared to POb, at embryonic and postnatal stages.

**Figure 6:**
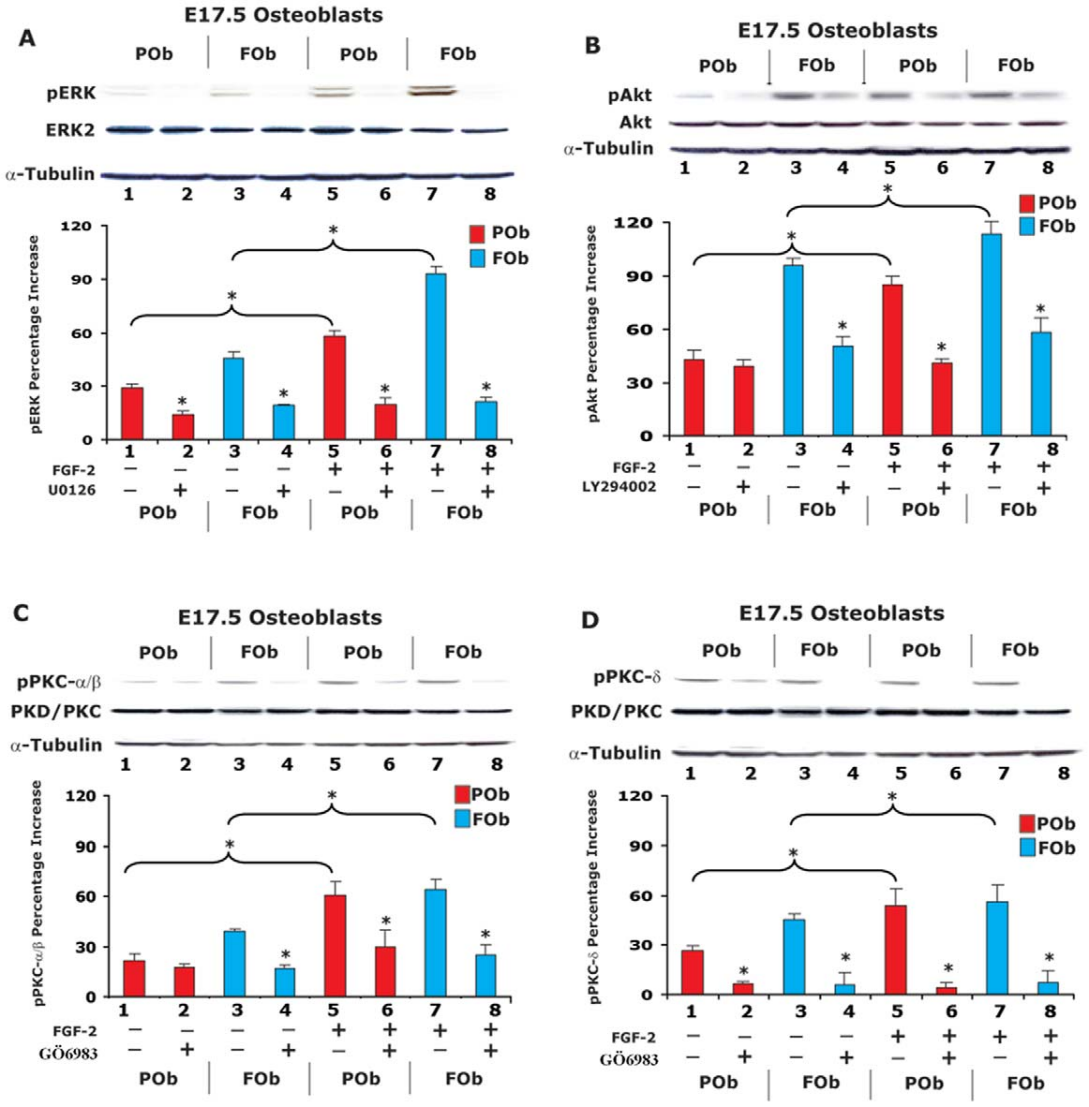
FGF-2 induction of the intracellular FGF signaling pathways on FOb and POb cells. FOb and POb cells harvested from E17.5 mice were cultured in serum free α-MEM for 12 hours in presence of specific inhibitors of the FGF signaling pathways to pre-empty endogenous FGF-2 activity. Then cells were treated with 20 ng/ml of rhFGF-2. **A**, effect of exogenous FGF-2 and inhibitors of MAPK signaling pathway. FGF-2 stimulation for 30 minutes increased phosphorylation of ERK protein equally in FOb and POb cells. Co-treatment with 10 µM U0126 inhibitor inhibited the FGF-2 induction as well as the endogenous phosphorylation of pERK protein in untreated FGF-2 FOb and POb cells. Histogram represents densitometric analysis of electrophoresis bands as above. **B**, effect of exogenous FGF-2 and inhibitors of PI3K signaling pathway. FGF-2 treatment resulted in increased pAkt phosphorylation which was two fold higher in POb cells compared to FOb cells. Treatment with 20 µM LY294002 inhibitor mostly abrogated the effect of exogenous FGF-2 and drastically reduced the endogenous level of pAkt protein in untreated FGF-2 FOb cells. Histogram represents densitometric analysis of electrophoresis bands. **C**, effect of exogenous FGF-2 and inhibitors of PKC α/β signaling pathway. FGF-2 treatment also induced an increased phosphorylation of PKC α/β proteins which was inhibited by co-treatment with 2 µM GÖ6983 inhibitor. Treatment with the inhibitor also reduced the endogenous level of p PKC α/β proteins in untreated FGF-2 cells. Histogram represents densitometric analysis of electrophoresis bands. **D**, effect of exogenous FGF-2 and inhibitors of PKC δ signaling pathway. FGF-2 and/or inhibitor treatments on PKC δ protein produced a similar effect to that observed on PKC α/β proteins. Asterisk * represents statistical significance (*p<0.05).

Next, we investigated the effect of either exogenous added FGF-2 protein or specific inhibitors of three FGF signaling pathways on both FOb and POb cells (**Figures 6A–D**). Stimulation with 20 ng/ml of rhFGF-2 increased phosphorylation of ERK by two folds in both FOb and POb cells (**Figure 6A**), whereas treatment with UO126 an inhibitor of MAP kinase pathway inhibited the effect of exogenous FGF-2, as well as, decreased the endogenous activation of ERK protein in FOb and POb cells (**Figure 6A**).

Stimulation with rhFGF-2 induced a stronger activation of PI3K, PKC α/β and δ pathways in POb cells as compared to FOb. Phosphorylated Akt, PKC α/β and δ proteins were two fold increased in POb cells as compared to FOb cells (**Figures 6B–D**). In parallel, the FOb and POb cells undergoing FGF-2 induction and their controls were also incubated with the two specific inhibitors LY294002 and G06983 to block the signaling pathways. The increased phosphorylation Akt, PKC α/β and δ proteins observed upon stimulation with exogenous rhFGF-2 was blocked by specific inhibitors, the treatment also decreased the endogenous levels of phosphorylated signaling proteins Akt and PKC α/β and δ. Moreover, the inhibitors treatment decreased the proliferation rate of both FOb and POb cells (Figure S1). Interestingly, treatment with UO126 an inhibitor of MAP kinase pathway decreased the proliferation rate of FOb cells to levels similar to that of untreated POb cells (Figure S1).

### Knockdown of FGF Receptor 1 abolishes the greater osteogenic potential of FOb cells, making them “POb like”

**Figure 7:**
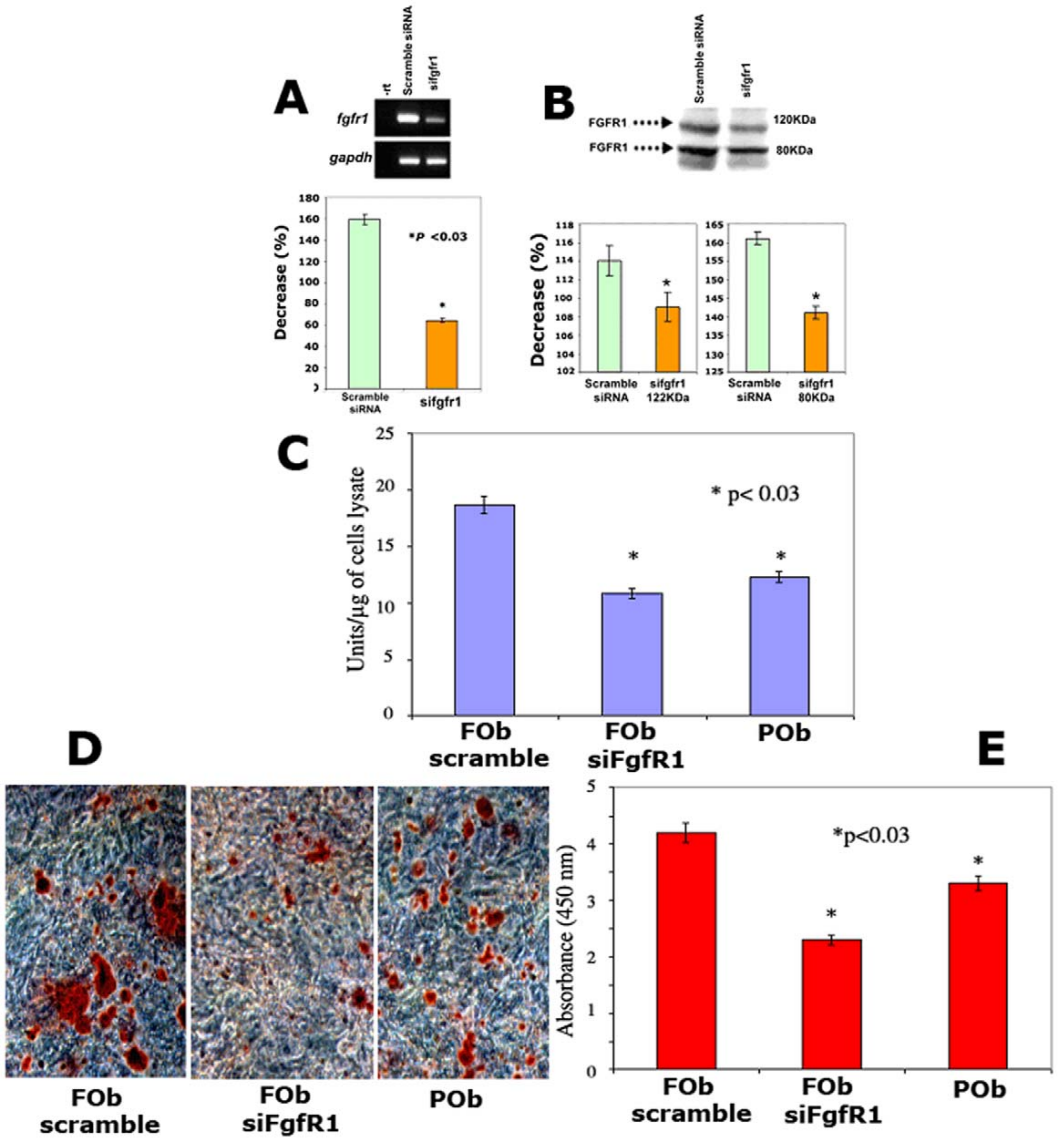
Silencing of FGF Receptor 1 abrogates the greater osteogenic potential of FOb cells. **A**, RT-PC analysis demonstrating a decrease in *fgfr1* gene expression by siRNA. Densitometry analysis of RT-PCR (Panel below), bands were scanned and quantified using Image J 1.36b software (NIH). **B**, Western blot demonstrating decreased levels of 2 different FGFR1 isoforms (80 kDa and 122 kDa) in si*FgfR1* transduced FOb cells. The intensity of the bands were quantified as above (Panels below). *p<0.05. **C**, Alkaline phosphatase enzymatic activity of si*FgfR*1 FOb cells demonstrating that frontal bone derived osteoblasts transduced with si*FgfR1* lost their greater osteogenic potential and became more POb-like. **D**, Alizarin red staining showing marked decreased of mineralization of extracellular matrix in si*FgfR1* FOb cells. **E**, Quantification of Alizarin Red. A lentivirus siRNA scramble was used as control.

Taken together the above data strongly indicated that FOb cells of neural crest origin were endowed with higher levels of FGF- 2 protein and endogenous activated FGF signaling compared to POb cells of mesodermal origin. These observations would suggest that activation of FGF signaling may play a crucial role in conferring the greater osteogenic potential to FOb cells. To determine the role of FGF signaling on the superior osteogenic potential displayed by frontal bone we permanently knocked-down *FgfR1* in FOb cells harvested from E 17.5 mice. In addition, we also compared the experimental and control FOb cells to untreated E17.5 POb cells. RT-PCR and immunoblotting analyses demonstrated that we could effectively knockdown the *Fgfr1* gene expression and the two FGFR1 isoform proteins (120 and 80 kDa) (**Figures 7A and B**). Osteogenic differentiation was monitored at an intermediate time point (day 10) by measuring the Alkaline phosphatase enzymatic activity. As shown in **Figure 7C**, at day 10 of differentiation si*FgfR1* osteoblasts displayed a significantly decreased Alkaline phosphatase enzymatic activity as compared to siRNA scramble FOb control cells. Interestingly, the level of Alkaline phosphatase activity in si*FgfR1* FOb cells became similar to that of POb cells. Subsequently, mineralization of extracellular matrix was assessed by Alizarin red staining at later time point (day 21) with the progression of differentiation. Quantification of Alizarin red staining revealed a significant decrease in mineralization of extracellular matrix of si*FgfR1* FOb cells as compared to siRNA scramble FOb control cells (**Figure 7D**). Thus, similarly to Alkaline phosphatase activity, Alizarin red staining also demonstrated a significant impaired osteogenic differentiation in si*FgfR1* FOb cells. Moreover, immunoblotting analysis of PCNA revealed decreased levels of protein in si*FgfR1* FOb cells, thus suggesting that downregulation of *FgfR1* effected also cell proliferation (data not shown).

## Discussion

Differences in the embryonic origin of mammalian bones composing the cranial vault have been well established [3]. While the frontal bones of the calvarium are neural crest in origin, the parietal bones arise from paraxial mesoderm. Our recent study demonstrated that frontal bones of both juvenile and adult mice are endowed with superior regenerative potential compared to parietal bones, and furthermore, osteoblasts derived from either juvenile or adult frontal bone display increased potential for proliferation and osteogenic differentiation when compared to osteoblasts obtained from the parietal bone [27]. Moreover, another study profiling the gene expression pattern of major pro-osteogenic FGF ligands and their receptors revealed substantial differences in the expression of specific FGF ligands such as *Fgf-2*, *-9* and *-18*, as well as, receptors *FgfR1*-, *R-2* and *R-3*, between neural crest derived frontal bone relative to paraxial mesoderm derived parietal bone [28]. Thus, the later study suggested that the frontal bone might represent an activated FGF domain as compared to parietal bone.

The current paper presents a follow up study addressing two main questions: first, how far back can the differences observed between frontal and parietal bones derived osteoblasts can be traced? Is the greater osteogenic potential of frontal bone derived osteoblasts a feature already detectable at embryonic stages or restricted to postnatal stages? Second, is the frontal bone really a domain with enhanced activation of FGF-signaling compared to parietal bone? The data gathered from the current study demonstrated that indeed striking differences in proliferation and osteogenic potential exist also between embryonic FOb and POb cells. Our results demonstrated a higher cell proliferation capability in neural crest derived osteoblasts compared to mesodermal osteoblasts. The higher mitogenic ability of FOb was also parallel by a greater potential for osteogenic differentiation.

To understand the molecular basis underlining the developmental differences of FOb and POb cells, we investigated the effect exerted by interactions between the two types of osteoblasts using co-culture and conditioned media methods. The results obtained from these experimental approaches demonstrated that FOb exerted a positive influence on cell proliferation, as well as, osteogenic differentiation, especially on POb cells, implicating that neural crest osteoblasts might express some pro-osteogenic molecules. Indeed, in agreement with what previously observed at gene expression level, our analysis revealed that FOb synthesized higher level of FGF-2 proteins and that the released FGF-2 was responsible at least in part for the greater proliferation rate of FOb. Of note, analysis for FGF-9 and FGF-18 proteins revealed also higher levels of these molecules in FOb as compared to POb cells (data not shown), thus further validating our previous data obtained from the gene expression profiling [28].

To further reveal molecular/signaling differences between FOb and POb cells in the context of FGF pathways, we investigated the extent of endogenous activation of the three major FGF signaling pathways, MAPK, PI3K, and PKC, in neural crest derived FOb and mesoderm derived POb. The endogenous phosphorylation of the signaling proteins were significantly higher in FOb than POb cells Differences between FOb and POb cells were especially prominent for the MAPK signaling pathway as indicated by more robust phosphorylation of ERK proteins in FOb cells. This observation supported the higher proliferative status of FOb cells compared to POb cells, as the role of MAPK pathway is well established in transducing the mitogenic activity elicited by FGF-2 [24], [31], [32].

Previous studies have linked PKC signaling pathways, especially PKC α/β and δ pathways to osteogenesis [25], [33]. In our study, the higher levels of phosphorylated PKC α/β and δ proteins observed in neural crest frontal osteoblasts nicely correlated with their greater osteogenic potential. Thus, confirming the involvement of FGF signaling in determining different osteogenic capacity between FOb and POb cells.

Other interesting findings were found for the PI3K pathway. Differences in the activation of signaling between FOb and POb cells were the most prominent among the three FGF signaling analyzed. Protein kinase B (PKB/Akt), a serine-threonine protein kinase and a downstream target of PI3K signaling pathway, has long been implicated in cell cycle progression, programmed cell death and cell survival [34], [35]. More recently, it has also been reported playing a crucial role in stem cell renewal [36].

Taking together, highly activated PI3K intracellular pathway with enhanced Akt phosphorylation activity may well reflect an increase in cell survival rate and cell renewal ability of neural crest derived FOb, indicating more progenitors entering the osteogenic differentiation pathway and less entering the apoptotic pathway within the frontal bone as compared to mesoderm derived parietal bone. This possibility could also explain the greater healing capacity of neural crest derived frontal bone compared to the mesoderm derived parietal bone [27]. The neural crest cells have been described as progenitor cells with higher cell renewal ability [37]. This may be one of the key features that differentiate neural crest osteoblasts from the mesenchymal osteoblasts. Our study revealed that differences between FOb and POb cells were more prominent at embryonic stages than early postnatal day 1. We have also observed that at later postnatal stages (P30 and P60), differences between FOb and POb cells in PI3K activation decreased, indicating that aging is also an important factor in controlling mitogenic and osteogenic potential of osteoblasts.

One of the interesting findings that emerged from this study was the inducible osteogenic potential of POb cells observed upon FGF-2 treatment. Addition of exogenous FGF-2 protein to POb cells made them “FOb-like”. Conversely downregulation of *FgfR1* in FOb cells impaired their osteogenic potential, mimicking POb cells. That opens the possibility to increase the proliferation and osteogenic potential of POb to that of FOb merely by treatment with exogenous growth factors or cytokines, (e.g. members of FGF family). Our results also suggested that the PI3K, PKC α/β and δ pathways might be more important for enhancing the plasticity of the mesenchymal parietal cells towards osteogenic differentiation. In addition, PI3K, PKC α/β and δ pathways might be more important for enhancing the plasticity of the mesenchymal POb cells towards osteogenic differentiation. Further exploration the molecular mechanisms and the interactions between the intracellular signaling pathways in neural crest derived osteoblasts would lead to the understanding of the osteogenesis in developing calvaria and the prospects of improving the differentiation stage-dependent mitogenesis and osteogenic differentiation in mesenchymal osteoblasts and aged osteoblasts.

We have recently reported, that the canonical Wnt-pathway has also an important role in determining the different osteogenic potential and healing capacity of frontal and parietal bones [27]. Moreover, FGF-2 treatment induces phosphorylation of GSK-3β and increases the nuclear levels of β-catenin in osteoblasts suggesting that enhanced activation of Wnt signaling might be mediated by FGF [27]. Thus, the data suggest that in frontal bone a cross-talk between FGF and canonical Wnt-signaling exists. In the light of our current and previous study we propose that FGF-ligands not only exert direct effects on osteoprogenitor cells but, may also have indirect effects by activating the canonical Wnt-signaling, during osteogenesis and/or bone regeneration.

Obtaining a comprehensive understanding of unique cell biology of calvarial osteoblasts from dissimilar embryonic origins, and understanding how those differences translate into variable potential strategies for endogenous bone regeneration, is critical to the optimization of cell-based skeletal tissue repair modalities.

## Supporting Information

Figure S1 Growth curve assay performed as described under Material and Methods section, on cells maintained in growth medium supplemented with or without the following inhibitors: 5 µM U-0126, 10 µM LY-294002 and 1 µM GÖ-6983. Control cells were maintained in growth medium containing 0.1%DMSO. The values are presented as means ± SD of three independent experiments.
(6.62 MB TIF)
