# Supplementary figures and images for: Activation of FGF Signaling Mediates Proliferative and Osteogenic Differences between Neural Crest Derived Frontal and Mesoderm Parietal Derived Bone
Source: PLoS One. 2010 Nov 18;5(11):e14033. doi: 10.1371/journal.pone.0014033 (PMC2987799; doi:10.1371/journal.pone.0014033)

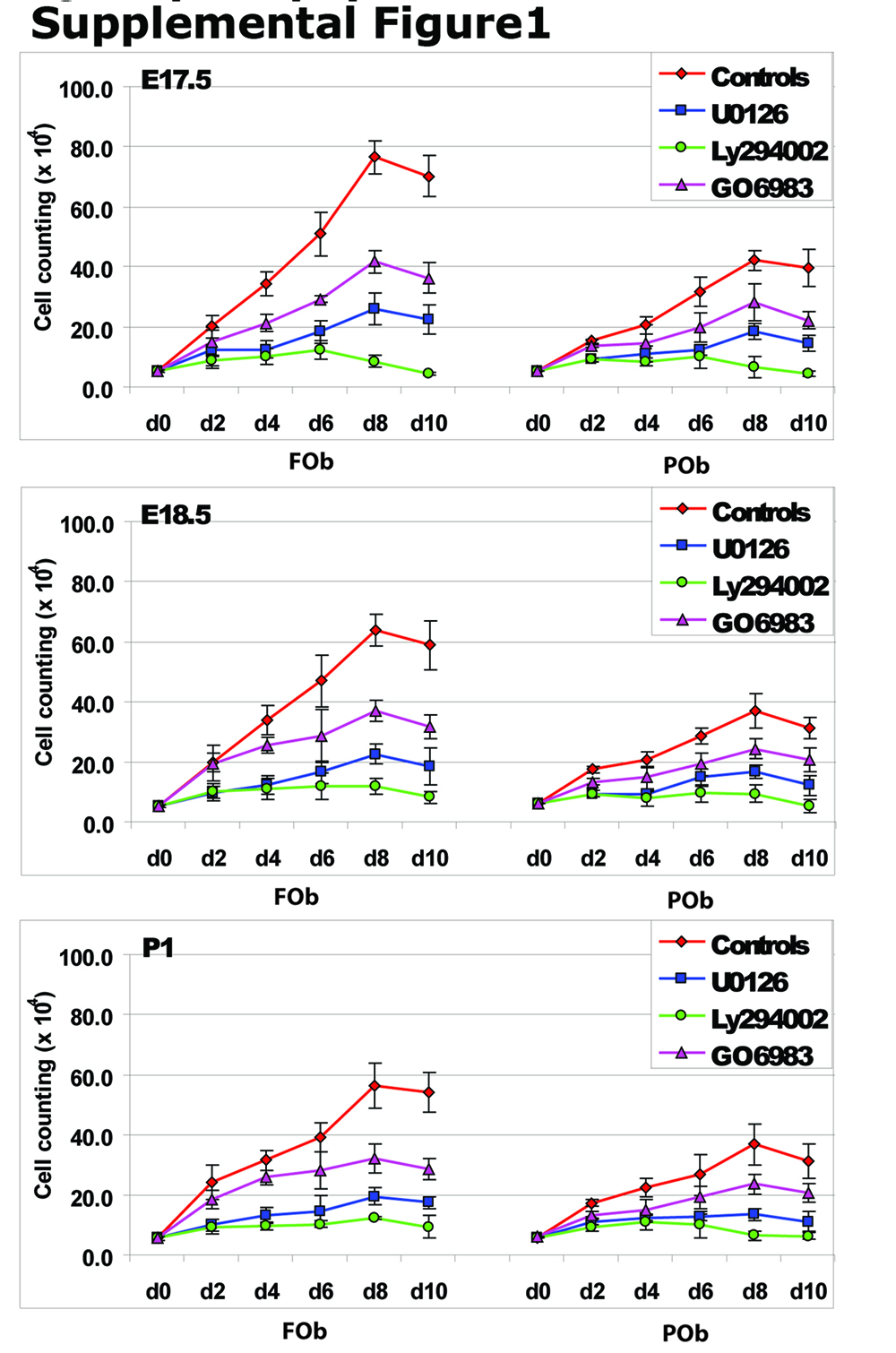

Supplement: Figure S1 — Growth curve assay performed as described under Material and Methods section, on cells maintained in growth medium supplemented with or without the following inhibitors: 5 µM U-0126, 10 µM LY-294002 and 1 µM GÖ-6983. Control cells were maintained in growth medium containing 0.1%DMSO. The values are presented as means ± SD of three independent experiments. (6.62 MB TIF) [file pone.0014033.s001.tif]
